# Sugar-sweetened beverage consumption and genetic predisposition to obesity in 2 Swedish cohorts^1^,^2^

**DOI:** 10.3945/ajcn.115.126052

**Published:** 2016-07-27

**Authors:** Louise Brunkwall, Yan Chen, George Hindy, Gull Rukh, Ulrika Ericson, Inês Barroso, Ingegerd Johansson, Paul W Franks, Marju Orho-Melander, Frida Renström

**Affiliations:** 3 Diabetes and Cardiovascular Disease—Genetic Epidemiology and; 4 Genetic and Molecular Epidemiology Unit, Department of Clinical Sciences, Lund University, Malmö, Sweden;; 5 National Institute for Health Research Cambridge Biomedical Research Centre and; 6 University of Cambridge, Metabolic Research Laboratories, Institute of Metabolic Science, Addenbrooke’s Hospital, Cambridge, United Kingdom;; 7 Wellcome Trust Sanger Institute, Cambridge, United Kingdom;; 8 Departments of Odontology,; 9 Public Health and Clinical Medicine, Section for Medicine, and; 10 Biobank Research, Umeå University, Umeå, Sweden; and; 11 Department of Nutrition, Harvard School of Public Health, Boston, MA

**Keywords:** sugar-sweetened beverage, genetic risk score, BMI, gene-lifestyle interaction, Sweden

## Abstract

**Background:** The consumption of sugar-sweetened beverages (SSBs), which has increased substantially during the last decades, has been associated with obesity and weight gain.

**Objective:** Common genetic susceptibility to obesity has been shown to modify the association between SSB intake and obesity risk in 3 prospective cohorts from the United States. We aimed to replicate these findings in 2 large Swedish cohorts.

**Design:** Data were available for 21,824 healthy participants from the Malmö Diet and Cancer study and 4902 healthy participants from the Gene-Lifestyle Interactions and Complex Traits Involved in Elevated Disease Risk Study. Self-reported SSB intake was categorized into 4 levels (seldom, low, medium, and high). Unweighted and weighted genetic risk scores (GRSs) were constructed based on 30 body mass index [(BMI) in kg/m^2^]-associated loci, and effect modification was assessed in linear regression equations by modeling the product and marginal effects of the GRS and SSB intake adjusted for age-, sex-, and cohort-specific covariates, with BMI as the outcome. In a secondary analysis, models were additionally adjusted for putative confounders (total energy intake, alcohol consumption, smoking status, and physical activity).

**Results:** In an inverse variance-weighted fixed-effects meta-analysis, each SSB intake category increment was associated with a 0.18 higher BMI (SE = 0.02; *P* = 1.7 × 10^−20^; *n* = 26,726). In the fully adjusted model, a nominal significant interaction between SSB intake category and the unweighted GRS was observed (*P-*interaction = 0.03). Comparing the participants within the top and bottom quartiles of the GRS to each increment in SSB intake was associated with 0.24 (SE = 0.04; *P* = 2.9 × 10^−8^; *n* = 6766) and 0.15 (SE = 0.04; *P* = 1.3 × 10^−4^; *n* = 6835) higher BMIs, respectively.

**Conclusions:** The interaction observed in the Swedish cohorts is similar in magnitude to the previous analysis in US cohorts and indicates that the relation of SSB intake and BMI is stronger in people genetically predisposed to obesity.

## INTRODUCTION

Easy access to energy-dense foods is a contributing factor to the ongoing global obesity epidemic. According to the National Health and Nutrition Examination Survey, the single largest source of added sugar in the US diet today comes from sugar-sweetened beverages (SSBs)^12^ (1). Epidemiologic studies, including clinical trials, have found SSB intake to be associated with obesity, cardiovascular disease, and type 2 diabetes (2, 3). There is to date no clear consensus about the relation between the consumption of artificially sweetened beverages (ASBs) and risk of obesity and cardiometabolic diseases (4, 5).

Obesity risk is also partly attributable to genetics, and although it is plausible that environmental factors might interact with genetic predisposition to modify the risk, only a few examples have so far been verified in independent cohorts (6). In a study by Qi et al. (7) that included 3 large prospective US cohorts of European ancestry, the association between SSB intake, but not ASB intake, and obesity risk was significantly more pronounced among participants with a high genetic predisposition to obesity.

The aim of this study was to replicate the interaction reported by Qi et al. and to investigate whether the risk of obesity associated with SSB (and ASB) intake is modified by common genetic predisposition to obesity in 2 large Swedish cohort studies of middle-aged participants: MDCS (Malmö Diet and Cancer Study) and GLACIER (Gene-Lifestyle Interactions and Complex Traits Involved in Elevated Disease Risk).

## METHODS

### Study participants and data collection

#### MDCS

MDCS is a prospective cohort study that was conducted in the city of Malmö in southern Sweden and has been described in detail elsewhere (8). All men born between 1923 and 1945 and all women born between 1923 and 1950 were invited via personal letters and advertisements in local newspapers and public places. Clinical characteristics, biomedical measures, and extensive information on lifestyle behaviors were collected (8), and baseline examinations were performed between 1991 and 1996. Participants with complete dietary data, genetic information, and without prevalent diabetes, cardiovascular disease, and cancer were eligible for this analysis (*n* = 21,824). All participants provided written informed consent, and the ethics committee at Lund University approved the MDCS protocols.

#### GLACIER

GLACIER is a population-based prospective cohort comprising ∼19,000 initially nondiseased adults living in the county of Västerbotten in northern Sweden nested within the Northern Sweden Health and Disease Study (9, 10). Clinical characteristics, biomedical measures, and extensive information on lifestyle behaviors were obtained as part of a population-wide health screening initiative called the Västerbotten Health Survey (also called the Västerbotten Intervention Program), in which habitants in the county of Västerbotten are invited to attend an extensive health examination the year of their 40th, 50th, and 60th birthdays (9). The total number of GLACIER participants with genotype and phenotype data available for this analysis was 4902, of whom the vast majority were born between 1932 and 1957. Baseline examinations were performed between 1991 and 2007. All participants provided written informed consent, and the regional ethical review board in Umeå approved all aspects of the study.

### Anthropometric measures

In both MDCS and GLACIER, weight was measured with the use of a calibrated balance-beam scale with participants wearing light clothes and no shoes. Height (to the nearest centimeter) was measured with a stadiometer, and BMI (in kg/m^2^) was calculated.

### Diet and lifestyle measurements

#### MDCS

Diet data were collected with a modified diet history method specifically designed for MDCS (11) that includes *1*) a 7-d menu booklet for registering cooked meals and cold beverages; *2*) a 168-item food-frequency questionnaire (FFQ), including portion sizes of regularly consumed foods not covered by the menu book; and *3*) a 45-min interview with additional questions about the cooking methods and product choices (conducted approximately 10 d after the clinical measurements were taken). A trained interviewer ensured that the reporting in the questionnaire and the 7-d menu booklet did not overlap. Mean daily food intake (g/d) was calculated based on the FFQ and 7-d menu booklet. The MDCS database, which mostly contains data from the PCKOST2-93 database from the National Food Administration in Uppsala, Sweden, was used to convert reported food intake into energy and nutrient information. A variable was created to indicate the season of data collection [winter (December–February), spring (March–May), summer (June–August), and fall (September–November)] and was included as a covariate in the analysis to adjust for potential seasonal confounding of the dietary intake. The diet assessment method was also included as a covariate and was defined as data collection before or after a minor change of coding routines implemented in September 1994.

Leisure-time physical activity was assessed by estimates of the number of minutes per week spent on 17 different activities. The duration was multiplied with an activity-specific intensity coefficient, and an overall leisure-time physical activity score was created (12, 13). Smoking status was defined as current smokers (including irregular smokers), former smokers, and never smokers. Based on the participant’s reported intake during the previous year, alcohol consumption was categorized as zero (no consumption), low (<15 g/d for women and <20 g/d for men), medium (15–30 g/d for women and 20–40 g/d for men), and high (>30 g/d for women and >40 g/d for men).

#### GLACIER

Information on diet was collected through a self-administered validated semiquantitative FFQ that was completed during the visit at the primary health care center (14). The FFQ initially covered 84 food items but was reduced in 1996 to 66 food items by combining several questions related to similar foods. The mean portion size of main protein sources (meat/fish), vegetables, and staple foods (potatoes/rice/pasta) were collected, and participants indicated how often they consumed various foods and beverages over the past year based on a 9-point frequency scale ranging from never to ≥4/d. Total energy intake was calculated based on the nutritional values available through the National Food Administration's database. Food intake level (total energy intake divided by estimated basal metabolic rate) was used to exclude participants with unreliable diet data. (The bottom 5% and top 1% of the food intake level distribution within the entire Västerbotten Health Survey population was excluded.) Alcohol consumption was quantified as g/d.

A modified version of the International Physical Activity Questionnaire was used to gather information on leisure-time physical activity for the past 3 mo categorized as never, occasionally (not regularly), 1–2 times/wk, 2–3 times/wk, or >3 times/wk. For the current analysis, categories were combined into a low (<1 time/wk) and a medium/high-leisure time physical activity level (≥1–2 times/wk). Smoking status was categorized as never, former, or current.

### Intake of SSB and ASB

#### MDCS

SSBs include all carbonated and noncarbonated beverages sweetened with energy-containing sweeteners (mainly sugar). Juice was not included. ASBs include all beverages with nonenergy artificial sweeteners such as sodas, pops, and other fruit drinks. One serving was defined as 250 g, and reported SSB and ASB intake was converted from g/d to servings/d and further categorized into 4 categories of intake: the first category contained individuals that did not report any SSB or ASB intake during the 7-d record (seldom consumers). The remaining participants were divided into tertiles of SSB intake (low, medium, and high). SSB and ASB intake were also dichotomized by combining the first 2 and last 2 categories.

#### GLACIER

In the initial FFQ version (covering 84 food items), SSB intake was covered by 2 questions—one on the intake of carbonated (sodas) and another on noncarbonated (e.g., nectar and syrups) SSBs that were combined into one variable for this analysis. After 1996, the FFQ was reduced, and the 2 questions covering SSB intake were combined into one that additionally included juice intake. In sensitivity analyses, excluding SSB intake assessed by the shorter FFQ (*n* = 1355) did not materially change how the results were interpreted. This analysis thus contained SSB intake assessed by both FFQ versions, and all models were additionally adjusted for a variable indicating FFQ version.

For this study, the 9-level frequency scale of the FFQ (never, occasionally, 1–3 times/mo, 1 time/wk, 2–3 times/wk, 4–6 times/wk, 1 time/d, 2–3 times/d, ≥4 times/d) was combined into 4 categories of intake (seldom, ≤2 times/y; low, 1–3 times/mo; medium, 1–3 times/wk; and high, ≥4 times/wk) to reflect the ranges within the 4 categories of SSB intake in MDCS. A dichotomized variable was constructed by combining the first 2 and last 2 categories (equivalent to ≤1–3 times/mo or ≥1–3 times/wk). No information on ASB consumption was available in GLACIER.

### Genotyping

#### MDCS

DNA was extracted from whole-blood samples with the use of Qiagen Maxipreps. Genotyping was performed with the use of a Sequenom MassArray matrix-assisted laser desorption/ionization-time-of-flight mass spectrometer that used Sequenom reagents and protocols. Single-nucleotide polymorphisms (SNPs) that failed Sequenom genotyping were genotyped individually with the use of TaqMan or KASPar allelic discrimination on an ABI 7900HT (Applied Biosystems) according to the manufacturer’s instructions. Of the 32 SNPs identified through genome-wide association study efforts to be associated with BMI (15), all but one (rs4836133 zinc finger protein 608) were present in MDCS, and 4 proxies (*r*^2^ > 0.8) were used (rs6548238 *THEM18*; rs17782313 melanocortin 4 receptor; rs7498665 SH2B adaptor protein 1; and rs10913469 SEC16 homolog B, endoplasmic reticulum export factor). The mean genotype call rate was 97.1%, and all SNPs were in Hardy-Weinberg equilibrium with *P* > 0.0016 (0.05/31). More details on the index and proxy SNPs and genotype quality control are listed in **Supplemental Table 1**.

#### GLACIER

DNA was extracted from peripheral white blood cells, and genomic DNA was diluted to 4 ng/μL (16). Genotyping was performed with the use of the MetaboChip array (Illumina Inc.). Of the 32 BMI-associated SNPs (15), 7 proxies were used that had an *r*^2^ > 0.8 (except rs1006353 mitochondrial translational initiation factor 3, which had an *r*^2^ = 0.74): rs10182181 Ras-associated protein Rap1; rs11165643 polypyrimidine tract-binding protein 2; rs1421085 fat mass and obesity-associated protein; rs7127684 ribosomal protein L27a; rs2030323 brain-derived neurotrophic factor; rs17109256 neurexin 3; and rs1006353 mitochondrial translational initiation factor 3. No proxy was available for the rs2890652 low-density lipoprotein receptor-related protein 1B locus. Genotyping success rate was >95%, and all 31 SNPs had a Hardy-Weinberg equilibrium with *P* > 0.0016 (0.05/31). More details on the index and proxy SNPs and genotype quality control are listed in Supplemental Table 1.

### Genetic risk score

To investigate aggregated genetic predisposition to obesity, a genetic risk score (GRS) was constructed based on the 32 BMI-associated loci. Missing genotypes were imputed in both cohorts for participants with >60% of the 32 BMI-associated loci successfully genotyped as previously described (17). In short, a missing genotype is replaced with the mean value for that SNP obtained from the fraction of participants with available genotyped data. Of the 32 BMI associated loci (15), rs4836133 zinc finger protein 608 and rs2890652 lipoprotein receptor-related protein 1B (or appropriate proxies) were not available in MDCS and GLACIER, respectively. To facilitate comparisons and subsequent meta-analyses of summary statistics, the GRSs were constructed based on the remaining 30 BMI-associated loci in both cohorts. A GRS was constructed for each participant by summing up the risk alleles at each of the 30 loci assuming an equal magnitude of effect at each locus. Genotypes were coded as 0, 1, and 2, indicating the number of BMI-associated alleles (15). In accordance with Qi et al. (7), a second GRS was constructed in which the contribution of each locus was weighted by its previously reported main effect on BMI (15) before being summarized into a weighted GRS (wGRS). To facilitate the interpretation and comparisons of results, the wGRS was transformed back to the same scale as the GRS by dividing each individual wGRS by the maximum possible wGRS score (8.46) and subsequently multiplying by 60 (the maximum number of risk alleles) (18). Although it is appropriate to take into consideration the contribution of each locus when constructing a GRS for main-effect analyses by weighting with previously established trait-specific effect sizes, there are thus far no good solutions for how to appropriately account for locus-specific contributions (i.e., main and interaction effects) in interaction analyses. For this reason, we present the results for the unweighted GRS throughout, and the results from the wGRS are presented in **Supplemental Table 2**. The results for both GRSs are similar overall.

### Statistical analysis

Generalized linear equations were used to model the effects of genotypes at each locus (coded as 0, 1, and 2) assuming an additive effect of alleles. The SSB variable was categorized into either 2 (seldom to low and medium to high) or 4 (seldom, low, medium, and high) rank-ordered categories. This variable was entered as a continuous variable in the models consistent with the analyses reported by Qi et al. (7). All models were adjusted for age-, sex-, and study-specific covariates as indicated. Interaction models included the product term (GRS × SSB or GRS × ASB) in addition to the marginal effect terms (GRS and SSB or ASB). To account for potential confounding factors, a second model was included that additionally adjusted for physical activity, smoking, alcohol consumption, and total energy intake. Total energy intake can be considered both a potential confounding and mediating factor in these analyses. Excluding total energy intake in sensitivity analyses did not materially change the results (data not shown) and was included as a covariate in the presented lifestyle-adjusted models. To illustrate the interaction, the association between the GRSs and BMI were analyzed by stratifying and categorizing SSB intake, and the association of SSB intake with BMI was stratified by cohort-specific quartiles of the GRSs. For MDCS, all analyses were done with the use of SPSS version 20.0 (IBM). SAS version 9.4 (SAS Institute) was used for GLACIER.

Cohort-specific effect estimates from main and interaction analyses and their respective variance estimates were combined with the use of inverse variance-weighted fixed-effects meta-analysis with the use of the *metan* command in Stata version 12 (StataCorp).

## RESULTS

### Marginal effect of SSBs, ASBs, and GRSs on BMI

Mean SSB daily intake in the overall cohort as well as within each of the 4 categories of intake was similar between MDCS and GLACIER (**Table 1**). In inverse variance-weighted fixed-effects meta-analyses, each increment in SSB intake category was associated with a 0.18 (SE = 0.02) higher BMI (*P* = 1.7 × 10^−20^; *n* = 26,726): β = 0.19 (SE = 0.02) per SSB category in MDCS (*P* = 1.2 × 10^−16^; *n* = 21,824) and β = 0.05 (SE = 0.06) per SSB category in GLACIER (*P* = 0.39; *n* = 4902). Analyses were adjusted for age-, sex-, and study-specific covariates and putative confounders (alcohol consumption, smoking status, physical activity, and total energy intake).

**TABLE 1:** Participant characteristics in the Swedish MDCS and GLACIER cohort studies^1^

		SSB intake^2^				
Characteristics	All	Seldom	Low	Medium	High	*P*-trend^3^
MDCS						
Participants, *n*	21,824	9865	4261	3775	3923	
Age, y	57.9 ± 7.7	58.2 ± 7.6	58.0 ± 7.8	57.2 ± 7.6	57.7 ± 7.7	<0.001
Sex, % women	62.1	63.1	66.2	63.5	53.8	<0.001
BMI, kg/m^2^	25.7 ± 3.8	25.6 ± 3.8	25.5 ± 3.7	25.7 ± 3.9	26.1 ± 4.0	<0.001
Alcohol intake, g/d	10.9 ± 12.7	11.3 ± 13.1	10.2 ± 11.1	11.1 ± 11.8	10.5 ± 13.3	<0.001
Current smoking, %	27.7	28.6	25.1	26.9	29.0	<0.001
Physically active, %	27.3	44.8	19.5	16.7	19.0	0.01
TEI, kJ/d	9460 ± 2562	9025 ± 2495	9393 ± 2440	9686 ± 2499	10,390 ± 2650	<0.001
SSB,^2^ servings/d	0.30 ± 0.58	0.00 ± 0.00	0.10 ± 0.07	0.36 ± 0.10	1.24 ± 0.83	<0.001
ASB, servings/d	0.04 ± 0.21	0.04 ± 0.22	0.04 ± 0.19	0.05 ± 0.20	0.05 ± 0.19	0.002
GRS,^4^ effect alleles	27.6 ± 3.4	27.7 ± 3.4	27.6 ± 3.4	27.5 ± 3.4	27.5 ± 3.4	0.13
GLACIER						
Participants, *n*	4902	781	1017	2238	866	
Age, y	49.0 ± 8.6	51.0 ± 8.1	51.2 ± 7.9	47.8 ± 8.7	47.7 ± 8.8	<0.0001
Sex, % women	62.0	77.5	69.0	57.3	51.9	<0.0001
BMI, kg/m^2^	25.5 ± 3.8	25.5 ± 3.8	25.6 ± 3.8	25.6 ± 3.8	25.5 ± 3.8	0.90
Alcohol intake, g/d	3.6 ± 4.4	2.8 ± 4.3	3.3 ± 4.0	3.9 ± 4.6	3.8 ± 4.7	<0.0001
Current smoking, %	21.6	25.1	23.2	19.5	22.1	0.01
Physically active, %	27.9	29.3	28.7	28.4	24.1	0.06
TEI, kJ/d	7455 ± 2407	6376 ± 1997	6787 ± 2152	7649 ± 2311	8703 ± 2582	<0.0001
SSB,^2^ servings/d	0.35 ± 0.50	0.004 ± 0.002	0.08 ± 0.002	0.26 ± 0.12	1.20 ± 0.67	<0.0001
GRS,^4^ effect alleles	27.5 ± 3.4	27.5 ± 3.2	27.6 ± 3.4	27.5 ± 3.4	27.4 ± 3.4	0.36

1 Data are means ± SDs unless otherwise indicated, *n* = 26,726. ASB, artificially sweetened beverage; GLACIER, Gene-Lifestyle Interactions and Complex Traits Involved in Elevated Disease Risk; GRS, genetic risk score; MDCS, Malmö Diet and Cancer Study; SSB, sugar-sweetened beverage; TEI, total energy intake.

2 For MDCS, the seldom-intake group included participants who did not report any intake of SSB (or ASB) in the 7-d menu booklet; the remaining participants were divided into a low-, medium-, and high-intake group based on tertiles of SSB (or ASB) intake. For GLACIER, ranges of intake were applied to reflect the 4 groups in MDCS.

3 ANOVA was used for continuous variables and the chi-square test for categorical variables.

4 The GRS comprises 30 BMI-associated single-nucleotide polymorphisms with a theoretical range from 0 to 60.

Each increment in category of ASB intake in MDCS was significantly associated with a 0.64 (SE = 0.04) higher BMI in MDCS (*P* = 3.9 × 10^−58^; *n* = 21,824). The model was adjusted for age-, sex-, and study-specific covariates and putative confounders as described previously. Adjusting additionally for SSB intake did not materially change the results (data not shown).

The GRS was significantly associated with BMI in both cohorts [MDCS: β = 0.09 (SE = 0.01) per allele, *P* = 5.5 × 10^−29^, *n* = 21,824; GLACIER: β = 0.16 (SE = 0.02) per allele, *P* = 2.5 × 10^−23^, *n* = 4902), analysis adjusted for age and sex. Weighting the GRS by accounting for previously reported SNP-specific effect estimates (15) yielded similar results [MDCS: β = 0.09 (SE = 0.01) per allele, *P* = 4.2 × 10^−10^, *n* = 21,824; GLACIER: β = 0.13 (SE = 0.01) per allele, *P* = 2.5 × 10^−21^, *n* = 4902)]. No association between the GRS and SSB (or ASB) intake was observed in either cohort (all *P* > 0.05). Cohort-specific marginal effects for the individual 30 BMI-associated loci are presented in **Supplemental Table 3**.

### Interaction between GRS, SSB, and ASB intake on BMI

As shown in **Table 2**, there was a significant interaction between the GRS and SSB intake [defined either as 4 categories (seldom, low, medium, and high intake) or dichotomized intake (seldom-to-low compared with medium-to-high intake)] on BMI in the pooled analysis. The magnitude of association of the GRS (per 10-unit increment) with BMI was greater with each SSB intake category increment (*P*-interaction = 0.02). Adjusting additionally for putative-confounding lifestyle factors slightly reduced the statistical significance of the observed interaction (*P*-interaction = 0.03) (Table 2). In the lifestyle-adjusted pooled analysis with dichotomized SSB intake, each 10-unit increment of the GRS was associated with a mean 1.31 (SE = 0.11) higher BMI in individuals reporting medium-to-high SSB intake (*P* = 1.2 × 10^−33^), equivalent to 3.8 kg in weight for a person 1.70 m tall. Among participants reporting seldom or low SSB intake, each 10-unit increment of the GRS was associated with a 0.83 (SE = 0.09) higher BMI (*P* = 6.0 × 10^−21^) or 2.4 kg in weight for a person 1.70 m tall (*P*-interaction = 0.01) (Table 2). Similar results were obtained with the wGRS (Supplemental Table 2). The effect modifications of the association between SSB intake (4 categories) and BMI by individual loci are presented in **Supplemental Table 4**.

**TABLE 2:** The association with BMI per 10 effect alleles of the GRS stratified by SSB and ASB intake in MDCS and GLACIER^1^

	Categories of intake					Categories of intake		
	Seldom	Low	Medium	High	*P*-interaction	Seldom to low	Medium to high	*P*-interaction
SSB								
MDCS								
Model 1	0.80 ± 0.12	0.69 ± 0.17	1.07 ± 0.18	1.03 ± 0.19	0.18	0.77 ± 0.09	1.05 ± 0.13	0.08
Model 2	0.83 ± 0.11	0.68 ± 0.16	1.06 ± 0.18	1.03 ± 0.19	0.28	0.79 ± 0.09	1.05 ± 0.13	0.12
* *GLACIER								
Model 1	0.61 ± 0.42	1.40 ± 0.33	1.71 ± 0.23	2.18 ± 0.38	0.003	1.10 ± 0.26	1.84 ± 0.20	0.02
Model 2	0.57 ± 0.42	1.40 ± 0.33	1.73 ± 0.23	2.30 ± 0.37	0.002	1.09 ± 0.26	1.88 ± 0.19	0.01
Pooled cohorts^2^								
Model 1	0.78 ± 0.11	0.83 ± 0.15	1.32 ± 0.14	1.26 ± 0.17	0.02	0.81 ± 0.09	1.30 ± 0.11	0.01
Model 2	0.82 ± 0.11	0.82 ± 0.15	1.32 ± 0.14	1.28 ± 0.17	0.03	0.83 ± 0.09	1.31 ± 0.11	0.01
ASB								
MDCS								
Model 1	0.85 ± 0.08	0.89 ± 0.48	0.95 ± 0.41	0.94 ± 0.56	0.63	0.81 ± 0.08	0.97 ± 0.33	0.58
Model 2	0.86 ± 0.08	0.98 ± 0.48	1.03 ± 0.41	0.92 ± 0.56	0.55	0.83 ± 0.08	1.01 ± 0.33	0.54

1 Data are β-coefficients ± SEs from linear regression models, *n* = 21,824 for MDCS and *n* = 4902 for GLACIER. Model 1 was adjusted for age-, sex-, and cohort-specific covariates. MDCS was additionally adjusted for season and method. GLACIER was adjusted for the food-frequency questionnaire version and the first 4 principal components. Model 2 includes Model 1 plus additional adjustments for physical activity, smoking status (current/former/never), alcohol consumption, and total energy intake. ASB, artificially sweetened beverage; GLACIER, Gene-Lifestyle Interactions and Complex Traits Involved in Elevated Disease Risk; GRS, genetic risk score; MDCS, Malmö Diet and Cancer Study; SSB, sugar-sweetened beverage.

2 Cohort-specific summary statistics were pooled with the use of an inverse variance-weighted, fixed-effects meta-analysis.

**FIGURE 1:**
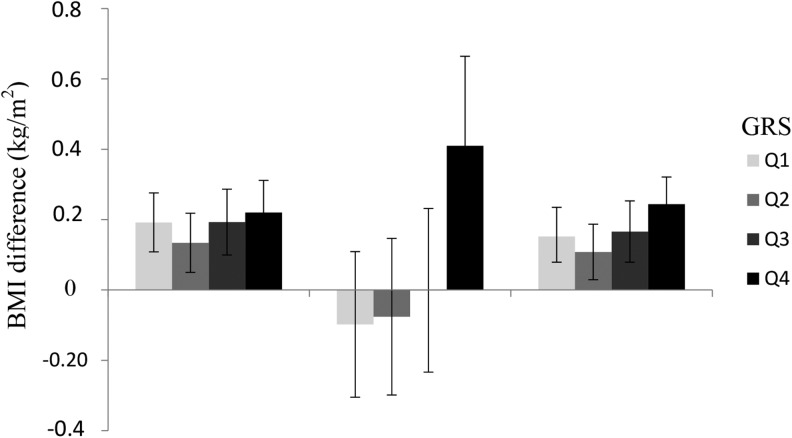
The difference in BMI (in kg/m^2^) associated with 1 increment in SSB intake (4 categories) in MDCS (*n* = 21,824), GLACIER (*n* = 4902), and a pooled analysis with the use of inverse variance-weighted fixed-effect meta-analysis stratified by quartiles (Q1–Q4) of the GRS. Data are β-coefficients and 95% CIs derived from linear regression models. Across the quartiles of the GRS, a 1-increment increase in SSB intake was associated with 0.19 (95% CI: 0.11, 0.28), 0.13 (95% CI: 0.05, 0.22), 0.19 (95% CI: 0.10, 0.29), and 0.22 (95% CI: 0.13, 0.31) increases in BMI in MDCS and with −0.10 (95% CI: −0.31, 0.11), −0.08 (95% CI: −0.30, 0.15), −0.001 (95% CI: −0.23, 0.23), and 0.41 (95% CI: 0.16, 0.66) changes in BMI in GLACIER. In the pooled analysis, 1 increment of SSB intake was associated with 0.15 (95% CI: 0.08, 0.24), 0.11 (95% CI: 0.03, 0.19), 0.17 (95% CI: 0.08, 0.25), and 0.24 (95% CI: 0.15, 0.32) changes in BMI (all *P* < 0.01). The analysis was adjusted for age, sex, alcohol consumption, smoking status, physical activity, total energy intake, and cohort-specific covariates. GLACIER, Gene-Lifestyle Interactions and Complex Traits Involved in Elevated Disease Risk; GRS, genetic risk score; MDCS, Malmö Diet and Cancer Study; SSB, sugar-sweetened beverage.

As illustrated in **Figure 1**, in pooled analyses the magnitude of the association between SSB intake (4 categories) and BMI increased by GRS quartile: each SSB category increment was associated with a 0.15 (SE = 0.04) higher BMI (*P* = 1.3 × 10^−4^; *n* = 6835) in the lowest quartile of the GRS compared with a 0.24 (SE = 0.04) (*P* = 2.9 × 10^−8^; *n* = 6766) higher BMI in the highest quartile—the mean difference equivalent to 266 g in weight for a person 1.70 m tall. Similar results were obtained with the wGRS (**Supplemental Figure 1**).

ASB, defined either by 4 levels or dichotomized intake, did not modify the association between the GRS and BMI in MDCS (Table 2), with similar results observed for the wGRS (Supplemental Table 2).

## DISCUSSION

In this study of 26,729 Swedish adults, we observed that the magnitude of the association between SSB intake and BMI is stronger in people genetically predisposed to obesity. Adjusting for potential confounding lifestyle factors had no material impact on these results. This finding is in agreement with the previously published findings by Qi et al. (7).

Many epidemiologic studies, including randomized intervention trials, have investigated the role of SSB in relation to obesity, weight maintenance, and BMI and have found strong associations with the consumption of SSB and increased BMI (1, 2, 19, 20). In contrast, a recent systematic review (21) concluded that the role of SSB intake in obesity risk remains unclear because many studies have not adjusted for total energy intake, which makes it difficult to evaluate whether SSB intake contributes to the risk of obesity beyond adding energy to the total diet. However, no intervention trials passed the inclusion criteria and were not included in the systematic review. The inconsistent results might also partly be explained by individual genetic susceptibility to obesity, as indicated by findings from 3 large US cohorts (7) and further corroborated in 2 Swedish cohorts in this study. Mean reported SSB intake SSB was similar between the US and Swedish cohorts.

ASB intake was available in MDCS and is more strongly associated with BMI than SSB. In accordance with Qi et al. (7), the association is not modified by genetic predisposition to BMI. The direction of causality between ASB and BMI has been much debated (22), and it is likely because of reverse causality that we observed such a strong association between ASB and BMI. However, a recent study in humans suggests that the constitution of the gut microbiota might mediate the association between the consumption of artificial sweeteners and glucose intolerance (23).

The main limitations of our study are the cross-sectional design and that data on SSB and ASB intake are self-reported. In addition, because the dietary assessment methods differ between the 2 cohorts, SSB intake could not be uniformly defined across the 2 cohorts. Although the results are more pronounced in GLACIER than MDCS, probably in part because of a larger proportion of participants being labeled as seldom consumers in MDCS as a result of the 7-d record period, the fact that we observed similar trends in both cohorts reduces the possibility that any aspect of the diet assessment method could have markedly confounded the results. The modified diet history method in MDCS is very detailed and collects data on current intake during the 7-d record period. The FFQ implemented in GLACIER was designed to capture mean food frequencies during the past year and is therefore more likely to capture habitual diet intake. These methodologic differences in diet assessment between MDCS and GLACIER result in differences in total energy intake between the 2 cohorts that might partly explain why we observed more of a dose-dependent effect modification in GLACIER, which is in line with the results observed in the US cohorts (7), whereas the results in MDCS indicate a threshold effect (Table 2). It is, however, important to keep in mind that seldom consumers in MDCS were individuals who did not report any intake of SSB (or ASB) during the specific 7-d recording period and might thus harbor a certain degree of misclassification.

In the United States, high-fructose corn syrup, a mixture of free glucose and fructose, is the caloric sweetener of sodas, whereas sucrose (a disaccharide made up of glucose and fructose) is the main sweetener used in Europe (19). It is still unclear whether there is a difference in the obesogenic effects of high-fructose corn syrup and sucrose, but the fact that we observed a similar interaction between genetic susceptibility of obesity and SSB on BMI in both the Swedish (albeit stronger in GLACIER than MDCS) and US cohorts implies that the underlying mechanism is independent of the type of sweetener.

Beyond the type of sweetener used in beverages, Sweden and the United States differ in many cultural and lifestyle aspects. This may even be true within Sweden given the great distance between north and south (1572 km). However, the results in the northern and southern Sweden cohorts are similar and in agreement with the observations in the US cohorts, supporting the original finding and suggesting that the interaction is likely to be generalizable to other populations of European ancestry.

In summary, there is a growing body of evidence from epidemiologic studies in support of the health benefits associated with an overall reduction in SSB consumption, and our results support the initial finding indicating that this may be even more relevant in people with a high genetic susceptibility to obesity.

## Footnotes

12 Abbreviations used: ASB, artificially sweetened beverage; FFQ, food-frequency questionnaire; GLACIER, Gene-Lifestyle Interactions and Complex Traits Involved in Elevated Disease Risk; GRS, genetic risk score; MDCS, Malmö Diet and Cancer Study; SNP, single nucleotide polymorphism; SSB, sugar-sweetened beverage; wGRS, weighted genetic risk score.
